# CD8^+^ T cells exhaustion induced by myeloid‐derived suppressor cells in myelodysplastic syndromes patients might be through TIM3/Gal‐9 pathway

**DOI:** 10.1111/jcmm.14825

**Published:** 2019-11-22

**Authors:** Jinglian Tao, Dong Han, Shan Gao, Wei Zhang, Hong Yu, Pei Liu, Rong Fu, Lijuan Li, Zonghong Shao

**Affiliations:** ^1^ Hematology Department Tianjin Medical University General Hospital Tianjin China; ^2^ Emergency Department Tianjin Medical University General Hospital Tianjin China

**Keywords:** CD8^+^ T cells, exhaustion, myelodysplastic syndrome

## Abstract

CD8^+^ T cells play a central role in antitumour immunity, which often exhibit ‘exhaustion’ in the setting of malignancy and chronic viral infection due to T cell immunoglobulin and mucin domain 3 (TIM3) and myeloid‐derived suppressor cells (MDSCs). Our team previously found that overactive MDSCs and exhausted TIM3^+^CD8^+^ T cells were observed in myelodysplastic syndromes (MDS) patients. However, it is not obvious whether MDSCs suppress CD8^+^ T cells through TIM3/Gal‐9 pathway. Here, Gal‐9, as the ligand of TIM3, was overexpressed in MDSCs. The levels of Gal‐9 in bone marrow supernatants, serum and culture supernatants of MDSCs from MDS patients were elevated. CD8^+^ T cells from MDS or normal controls produced less perforin and granzyme B and exhibited increased early apoptosis after co‐culture with MDSCs from MDS. Meanwhile, the cytokines produced by CD8^+^ T cells could be partially restored by TIM3/Gal‐9 pathway inhibitors. Furthermore, CD8^+^ T cells produced less perforin and granzyme B after co‐culture with excess exogenous Gal‐9, and the function of CD8^+^ T cells was similarly restored by TIM3/Gal‐9 pathway inhibitors. Expression of Notch1, EOMES (associated with perforin and granzyme B secretion), p‐mTOR and p‐AKT (associated with cell proliferation) was decreased in CD8^+^ T cells from MDS after co‐culture with excess exogenous Gal‐9. These suggested that MDSCs might be the donor of Gal‐9, and TIM3/Gal‐9 pathway might be involved in CD8^+^ T cells exhaustion in MDS, and that TIM3/Gal‐9 pathway inhibitor might be the promising candidate for target therapy of MDS in the future.

## 1 INTRODUCTION

Myelodysplastic syndromes (MDS) are malignant bone marrow disorders characterized by ineffective haematopoiesis and high risk to transformation into acute myeloid leukaemia. Increased malignant clones and cellular immunodeficiency might contribute to the pathogenesis of MDS.^1^, ^2^, ^3^, ^4^, ^5^ Cytotoxic T lymphocytes (CTLs) play a central role in tumour immunity.^6^ Adoptive T cell immunotherapy in neoplastic disorders has been restricted by CD8^+^ T cells ‘exhaustion’ due to some immune checkpoint inhibitors, such as T cell immunoglobulin and mucin domain 3 (TIM3), programmed death‐1 (PD‐1), cytotoxic T lymphocyte antigen 4 (CTLA‐4) and lymphocyte‐activation gene 3 (LAG3).^7^, ^8^

Among these immune checkpoints, the interaction of TIM3 with its ligand galectin‐9 (Gal‐9) promotes Tc1 cells^9^ apoptosis, CD8^+^T cells exhaustion,^10^, ^11^ malignant cell proliferation^12^ and myeloid‐derived suppressor cells (MDSCs) amplification.^13^ In humans, MDSCs are usually defined as CD14^−^CD11b^+^ cells or the cells which express the myeloid marker (CD33) and are lack of lineage maturation markers and HLA‐DR^14^ (Lin^−^HLA‐DR^−^CD33^+^ cells). In previous research, increased Lin^−^HLA‐DR^−^CD33^+^ MDSCs in MDS patients were observed, and this cell population was obviously separated by flow cytometry than other labelled cells. We also found that CD8^+^ T cells could be inhibited by MDSCs, which were ‘crazy’ in MDS patients. CD8^+^ T cells with less INF‐γ overexpressed TIM3 in MDS patients. Further, TIM3^+^CD8^+^ T cells produce less perforin and granzyme B.^1^, ^2^ The hypothesis that CD8^+^ T cells exhaustion might be induced by MDSCs through TIM3/Gal‐9 pathway drove us to detect Gal‐9 expression in MDSCs, and the level of perforin and granzyme B in CD8^+^ T cells after co‐culture with MDSCs or excess exogenous Gal‐9 or TIM3/Gal‐9 pathway inhibitors. We also explored Notch1, eomesodermin (EOMES), phospho‐mTOR (p‐mTOR) and phospho‐AKT (p‐AKT) proteins, which are related to the function and proliferation of CD8^+^ T cells,^15^, ^16^ to verify that TIM3/Gal‐9 pathway might be involved in CD8^+^ T cells exhaustion induced by MDSCs in MDS patients.

## 2 MATERIALS AND METHODS

### 2.1 Patients

Fifty‐two treatment‐naive Chinese MDS patients (32 males and 20 females, median age 64, Table 1) who were newly diagnosed according to the WHO classification^17^ in the Hematology Department of Tianjin Medical University General Hospital from January 2017 to August 2019 were enrolled, including patients with MDS with single lineage dysplasia (MDS‐SLD, n = 4), MDS with multilineage dysplasia (MDS‐MLD, n = 4), MDS‐RS (MDS with ring sideroblasts) with single lineage dysplasia (MDS‐RS‐SLD, n = 3), MDS‐RS with multilineage dysplasia (MDS‐RS‐MLD, n = 5), MDS with isolated del (5q) (n = 2), MDS with excess blasts 1 (MDS‐EB‐1, n = 20), MDS with excess blasts 2 (MDS‐EB‐2, n = 13) and MDS unclassifiable (MDS‐U, n = 1). Thirty‐one healthy volunteers (mean age 44.48 ± 14.87) were enrolled. There was no difference in the sex ratio or age between MDS patients and normal healthy volunteers (*P* > .05).

**Table 1:** Clinical features of MDS patients were studied here

Sample	Sex	Age	WHO subtype	Blasts of BM (%)	Cytogenetics	IPSS
001	Female	61	MDS‐EB‐1	6	7p‐,18p‐	2
002	Male	65	MDS‐MLD	1	(‐)	0.5
003	Male	55	MDS‐EB‐2	16.5	(‐)	2
004	Male	67	MDS‐U	1	‐7	1.5
005	Male	68	MDS‐EB‐2	14.5	8q‐,‐5,‐7	3
006	Female	29	MDS‐MLD	1	(‐)	0
007	Female	52	MDS‐EB‐1	5	(‐)	0
008	Male	66	MDS‐RS‐MLD	0.5	(‐)	0.5
009	Female	74	MDS‐EB‐2	17	5q‐,7q‐,+8	3
010	Male	63	MDS‐EB‐1	7	8q‐	1.5
011	Female	65	MDS‐EB‐1	5.5	5q‐,7q‐,+8	2
012	Male	68	MDS‐MLD	4	7q‐,‐7	1.5
013	Male	35	MDS‐RS‐SLD	1	(‐)	0
014	Female	63	MDS‐EB‐1	7.5	(‐)	1
015	Female	65	MDS with del (5q)	0.5	5q‐,+8	0.5
016	Female	52	MDS‐EB‐2	16	(‐)	2.0
017	Female	72	MDS‐RS‐MLD	0	(‐)	0.5
018	Male	30	MDS‐EB‐1	7.5	20q‐	2.0
019	Male	64	MDS‐EB‐2	11	(‐)	2.0
020	Male	60	MDS‐EB‐1	5.5	(‐)	1
021	Male	54	MDS‐EB‐2	19	+8	2.5
022	Male	52	MDS‐EB‐2	12	(‐)	2
023	Male	61	MDS‐EB‐1	9	(‐)	1.0
024	Female	45	MDS‐EB‐1	6.5	+8	2.0
025	Male	26	MDS‐SLD	1	(‐)	0
026	Female	66	MDS‐MLD	0	(‐)	0.5
027	Male	52	MDS‐EB‐2	11	(‐)	2
028	Female	48	MDS‐EB‐2	16	(‐)	2
029	Male	64	MDS‐EB‐1	5	(‐)	1
030	Male	76	MDS‐EB‐1	5	(‐)	1.0
031	Male	59	MDS‐EB‐1	9	(‐)	1.0
032	Male	55	MDS‐EB‐1	5	(‐)	1.0
033	Female	59	MDS‐EB‐1	8	t(2;12)(p12;p12)	1.5
034	Female	67	MDS‐EB‐1	6.5	(‐)	1.0
035	Female	68	MDS‐RS‐MLD	4	(‐)	0.5
036	Male	84	MDS‐RS‐MLD	0	(‐)	0.5
037	Male	65	MDS‐EB‐1	5.5	(‐)	1
038	Male	44	MDS‐SLD	1	(‐)	0
039	Male	64	MDS‐EB1	5.5	(‐)	1
040	Male	71	MDS‐RS‐MLD	3	7q‐	1.5
041	Male	39	MDS‐EB1	7	(‐)	0.5
042	Female	67	MDS‐RS‐SLD	2	(‐)	0
043	Male	62	MDS‐EB2	16	(‐)	2
044	Male	69	MDS‐SLD	3	7q‐,20q‐	1.5
045	Female	66	MDS‐EB2	18	5q‐,20q‐	2
046	Male	68	MDS‐RS‐SLD	1	7q‐	1
047	Female	73	MDS‐EB1	7	(‐)	1
048	Male	65	MDS‐EB2	15.5	(‐)	2
049	Male	74	MDS‐EB1	9	(‐)	1
050	Male	62	MDS‐EB2	16	(‐)	2
051	Female	81	MDS with del (5q)	1	5q‐	1
052	Female	59	MDS‐SLD	0	‐7	1.5

The study was approved by the Ethics Committee of Tianjin Medical University General Hospital. Informed written consents were obtained from all the patients and normal controls following the Declaration of Helsinki.

### 2.2 Flow cytometry analysis

#### 2.2.1 Membrane markers and intracellular markers detection

One hundred microlitres of peripheral blood and bone marrow samples from MDS patients and healthy volunteers were placed in heparin‐containing anticoagulant tubes. The specimens were incubated with 15 µL of Lin/HLA‐DR/CD33 and their isotype control antibodies at 4°C for 30 minutes. After incubation, erythrocytes were lysed with 2 mL of erythrolysin for 10 minutes, centrifuged at 400 *g* for 5 minutes and washed twice with phosphate buffered saline (PBS). After permeabilizing the cell membrane using an IntraSure Kit (BD Biosciences), 5 μL galectin‐9 monoclonal antibody was added to the cells, incubated for 20 minutes at 4°C in the dark and washed twice with PBS. Finally, 5 × 10^5^ cells per tube were detected by flow cytometry. After CD8^+^ T cells and MDSCs were co‐cultured, they were co‐incubated with CD3/CD8 antibodies in the same way. For intracellular staining, the samples were incubated with perforin and granzyme B antibodies after permeabilizing the cell membrane. The phenotype of MDSCs was analysed for the cell surface markers Lin, HLA‐DR and CD33. Intracellular expression of galectin‐9 was determined. Perforin or granzyme B expression in co‐cultured CD8^+^ T cells was analysed. All data were collected on a flow cytometer (Beckman Coulter), and the results were analysed with Kaluza software (Beckman Coulter).

The labelled antibodies included CD3‐APC (SK7, BD Biosciences), CD3‐PE (SK7, BD Biosciences), CD8‐FITC (SK1, BD Biosciences), TIM3‐APC (7D3, BD Biosciences), Lin‐FITC (BD Biosciences), HLA‐DR‐PerCP (L243, BD Biosciences), CD33‐APC (WM53, BD Biosciences), galectin‐9‐PE (9M1‐3, BD Biosciences), perforin‐PE (δG9, BD Biosciences) and granzyme B‐PE (GB11, BD Biosciences). The above antibodies were added as described by the manufacturer.

#### 2.2.2 Detection of apoptosis

An apoptosis assay (FITC Annexin V Apoptosis Detection Kit I, BD Biosciences) was used to detect apoptosis of CD8^+^T cells co‐cultured with MDSCs. The cells were washed twice with cold PBS and were resuspended in 1× binding buffer at a concentration of 1 × 10^6^ cells/mL. Then, 100 µL of the solution (1 × 10^5^ cells) was transferred to a 5‐mL culture tube, and 5 µL of FITC Annexin V and 5 µL of PI were added. The cells were gently vortexed and incubated for 15 minutes at room temperature (25°C) in the dark. Finally, 400 µL of 1× binding buffer was added to each tube. Analysis was performed by flow cytometry (Beckman Coulter).

### 2.3 Sorting CD8^+^ T cells and MDSCs

Ten millilitres of fresh peripheral blood or bone marrow was obtained from MDS patients or normal controls (NC). Human CD8^+^ immunomagnetic bead solution (Miltenyi Biotec) (50 µL) was added to mononuclear cells, which were obtained by Ficoll gradient centrifugation. The samples were incubated for 15 minutes at 4°C and washed once with buffer, and the suspension cells were passed through the MS column in the magnetic field. Then, the column was removed from the magnet and 1 mL of wash buffer was added to the top of the column and the plunger (in the same package as the column) was immediately used to force the buffer through the column. The collected cells were used for the subsequent experiments.

Ten millilitres of bone marrow was obtained from MDS patients, and red blood cells were lysed with lysing solution (BD Biosciences) and washed with PBS; and then, 40 µL of Lin\HLA‐DR\CD33 antibodies were added to label the surface markers of the MDSCs. The cells were co‐incubated for 30 minutes at 4°C in the dark and washed with PBS. The cells were collected by the FACS Aria II (BD Biosciences).

### 2.4 Cells culture

#### 2.4.1 Culture MDSCs

MDSCs were sorted by flow cytometry and then cultured with 10% foetal bovine serum (FBS) (containing 60 mg/L penicillin and 100 mg/L streptomycin) (Gibco) in the presence of 50 ng/mL recombinant human (rh) granulocyte‐monocyte colony‐stimulating factor (GM‐CSF) (PeproTech Inc) at 37°C in a 5% CO_2_ incubator. The culture supernatants were collected after 48 hours for ELISA.

#### 2.4.2 Co‐culture of CD8^+^ T cells with MDSCs

To investigate whether MDSCs affect the function of CD8^+^ T cells via the TIM3/Gal‐9 pathway, 10^5^ CD8^+^ T cells and MDSCs (>95% Lin^−^HLA‐DR^−^CD33^+^ cells) at a ratio of 1:2 were supplemented with 10% foetal bovine serum (FBS) (containing 60 mg/L penicillin and 100 mg/L streptomycin) and co‐cultured at 37°C in a 5% CO_2_ incubator for 24‐48 hours. The effect of MDSCs on CD8^+^ T cells was analysed by adding 10 µg/mL TIM3 inhibitor (F38‐2E2, BioLegend) and 10 µg/mL Gal‐9 inhibitor (9M1‐3, BioLegend). The expression of perforin and granzyme B in CD8^+^ T cells was detected by flow cytometry, and the apoptosis of CD8^+^ T cells was assessed by using an Apoptosis Kit (BD Biosciences).

#### 2.4.3 Co‐culture of CD8^+^ T cells with exogenous Gal‐9

CD8^+^ T cells selected by magnetic beads were co‐cultured with 2 ng/mL exogenous recombinant human Gal‐9 (R&D Systems) with 10% FBS (containing 60 mg/L penicillin and 100 mg/L streptomycin) at 37°C in a 5% CO_2_ incubator for 24‐48 hours. Then, the expression of perforin, granzyme B and pathway‐related proteins in CD8^+^ T cells were detected with or without TIM3/Gal‐9 inhibitors.

### 2.5 Proliferation assay

CD8^+^ T cells from normal controls were stained with 2.5 μmol/L carboxyfluorescein diacetate succinimidyl ester (CFSE, BD Biosciences) for 10 minutes in a 37°C water bath and washed with 9× the original volume of PBS. The cells were pelleted by centrifugation, the supernatant was decanted, and 10 mL of complete medium with 10% FBS was added to stop the reaction. CFSE‐labelled CD8^+^ T cells were cultured with 1 µg/mL anti‐CD3 (OKT, eBioscience), 1 µg/mL anti‐CD28 (CD28.2, eBioscience) and 20 U/mL rhIL‐2 (R&D Systems) with or without MDSCs from MDS. The proliferation of CD8^+^ T cells in NC was determined by measuring CFSE fluorescence using flow cytometry.

### 2.6 Real‐time PCR

Expression of the Gal‐9 gene in MDSCs was detected by real‐time quantitative PCR. Purified MDSCs from MDS patients and normal controls were lysed using TRIzol reagent (Invitrogen Life Technologies). RNA was reverse‐transcribed using a DNA Synthesis Kit according to the manufacturer's protocol (TAKARA). The primers are listed in Table 2. Reactions were performed using the Bio‐Rad iQ5 Real‐Time System and the respective SYBR Green I kit (TAKARA). The mRNA level of Gal‐9 was normalized to that of *β*‐actin, and the relative expression of mRNA was calculated by the 2^−ΔΔCt^ method.

**Table 2:** Primer sequence of Gal‐9 and *β*‐actin in PCR

Target gene	Primer sequences	Annealing temperature (°C)
Gal‐9	F:5′‐CGGTTTGAAGATGGAGGGTA‐3′ R:5′‐CAGGAAGCAGAGGTCAAAGG‐3′	59
*β*‐Actin	F:5′‐TTGCCGACAGGATGCAGAA‐3′ R:5′‐GCCGATCCACACGGAGTACT‐3′	56

Abbreviations: F, forward primer; R, reverse primer.

### 2.7 Enzyme‐linked immunosorbent assay (ELISA)

Bone marrow supernatants, serum and culture supernatants of MDSCs from MDS patients and normal controls were harvested. The levels of Gal‐9 were measured by using a Human Galectin‐9 ELISA Kit (KAMIYA Biomedical Company) according to the manufacturer's instructions.

### 2.8 Western blot analysis

CD8^+^ T cells cultured alone and co‐cultured with exogenous Gal‐9 were collected and lysed in RIPA buffer supplemented with complete protease inhibitors and phosphatase inhibitors (Roche). Protein levels were quantified using a BCA Kit. Proteins were separated by electrophoresis on 4%‐12% precast gels (Bio‐Rad Laboratories) and transferred to nitrocellulose membranes (Millipore Corp). The membranes were blocked in 5% skimmed milk (BD Biosciences) or 5% bovine serum albumin (BSA) (Solarbio) and then incubated overnight at 4°C with primary antibodies against Notch1, p‐mTOR, p‐AKT, EOMES and GAPDH. After washing the membranes 3 times in TBST (Tris‐HCl containing NaCl and Tween 20), the membranes were incubated with the relevant horseradish peroxidase–conjugated secondary antibodies (1:5000 dilution, Cell Signaling Technology). A summary of the primary antibodies mentioned above is provided in Table 3. The reaction was detected using Super ECL Plus Detection Reagent. Protein levels were normalized to GAPDH.

**Table 3:** Primary antibodies

Antibodies	Species	Type	Dilution	Source
Notch1	Rabbit	Monoclonal IgG	1:1000	CST
p‐mTOR	Rabbit	Monoclonal IgG	1:1000	CST
p‐AKT	Rabbit	Monoclonal IgG	1:2000	CST
EOMES	Rabbit	Monoclonal IgG	1:1000	CST
GAPDH	Rabbit	Monoclonal IgG	1:1000	CST

Abbreviations: CST, Cell Signaling Technology (Danvers, MA, USA); EOMES, eomesodermin; p‐AKT, phospho‐AKT; p‐mTOR, phospho‐mTOR.

### 2.9 Statistical analysis

Prism statistical software (v7.00; GraphPad Software, Inc, La Jolla, CA, USA) was applied for data analysis. Statistical differences between two groups were analysed using Student's *t* test. One‐way ANOVA was used to compare three groups or more. All data are presented as the mean ± standard error of the mean (normal distribution data) or median (25% percentile‐75% percentile) (non‐normal distribution data).

## 3 RESULTS

### 3.1 Galectin‐9 was highly expressed in MDSCs, and Gal‐9 was also elevated in bone marrow supernatants, serum and culture supernatants of MDSCs from MDS patients compared to those from normal controls

**Figure 1:**
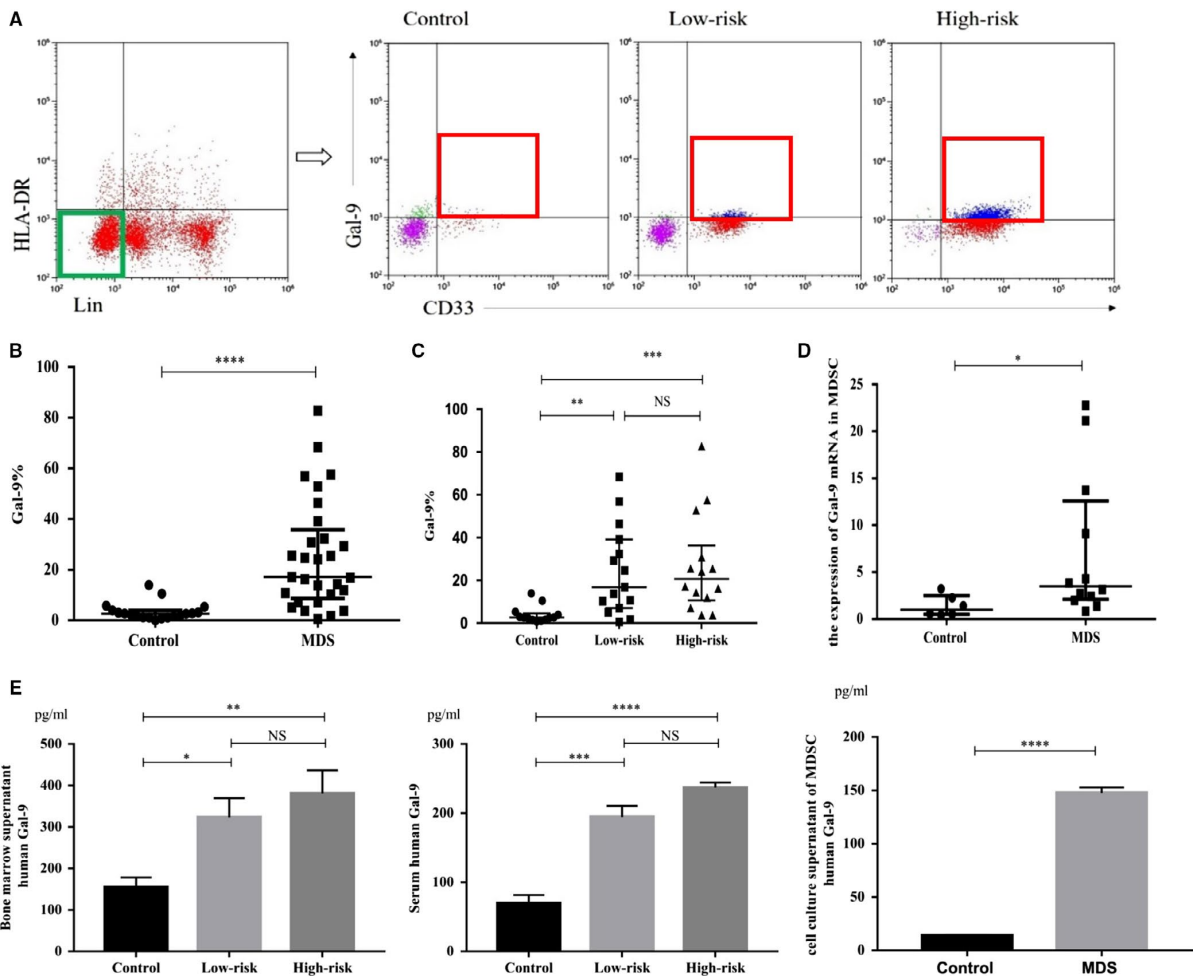
The expression of Gal‐9 in MDSCs. (A) The cells in the green rectangle are Lin^−^HLA‐DR^−^ cells, and the cells in the red rectangle are Gal‐9^+^Lin^−^HLA‐DR^−^CD33^+^ cells. Representative flow cytometry scatter diagrams of Gal‐9 expression in Lin^−^HLA‐DR^−^CD33^+^ cells are shown in control, low‐risk or high‐risk MDS patients. UR upper right quadrant, LR lower right quadrant. Gal‐9^+^Lin^−^HLA‐DR^−^CD33^+^ cells/Lin^−^HLA‐DR^−^CD33^+^ cells = UR/(UR + LR). (B and C) The dot diagrams present the percentage of Gal‐9^+^Lin^−^HLA‐DR^−^CD33^+^ cells/Lin^−^HLA‐DR^−^CD33^+^ cells in MDS patients (including low‐risk and high‐risk MDS patients) and in controls. (D) Gal‐9 mRNA in MDSCs by quantitative RT‐PCR. (E) Gal‐9 in bone marrow supernatants, serum and MDSC culture supernatants. *P < .05, **P < .01, ***P < .001, ****P < .0001, NS = not significant

Gal‐9 is highly expressed on tumour cells and Th1 cells. The expression of Gal‐9 in MDSCs in MDS is still not obvious. Therefore, we measured the expression of Gal‐9 in MDSCs (Lin^−^HLA‐DR^−^CD33^+^ cells) by using flow cytometry and RT‐PCR. The median percentage of Gal‐9 in MDSCs from MDS patients (n = 29, samples: 001‐031, except 005 and 018) was significantly higher than that in NC (17.21% (8.74%‐35.77%) vs 2.61% (1.19%‐3.39%), *P* < .0001****), but there was no difference between the low‐risk and high‐risk groups (16.86% (7.15%‐39.19%) vs 20.72% (10.74%‐36.35%), *P* = .7148) (Figure 1A‐C). Gal‐9 mRNA in MDSCs from MDS patients (n = 12, samples: 001, 004‐007, 016, 018, 024, and 031‐034) was higher than that in NC (3.49 (2.09‐12.57) vs 0.99 (0.50‐2.50), *P* < .05*) (Figure 1D).

The levels of Gal‐9 in bone marrow supernatants, serum (n = 15, samples: 038‐052) and culture supernatants of MDSCs (n = 3, samples: 048, 049, 050) from both low‐risk and high‐risk MDS were significantly higher than those in NC (bone marrow supernatant: 323.6 ± 42.6 pg/mL vs 381 ± 55.98 pg/mL vs 154.9 ± 23.72 pg/mL, *P* = .0042**; serum: 194.5 ± 16.13 pg/mL vs 237.1 ± 7.18 pg/mL vs 69.81 ± 11.93 pg/mL, *P* < .0001****; culture supernatants of MDSCs: 147.7 ± 5.24 pg/mL vs 14.2 ± 0.55 pg/mL, *P* < .0001****) (Figure 1E), but there was no difference between the low‐risk and high‐risk groups. These data suggested that MDSCs might release Gal‐9 into the bone marrow microenvironment and exert immunosuppressive effects.

### 3.2 Perforin, granzyme B and proliferation of CD8^+^ T cells in normal controls decreased after co‐cultivation with MDSCs, while the early apoptosis of CD8^+^ T cells increased

**Figure 2:**
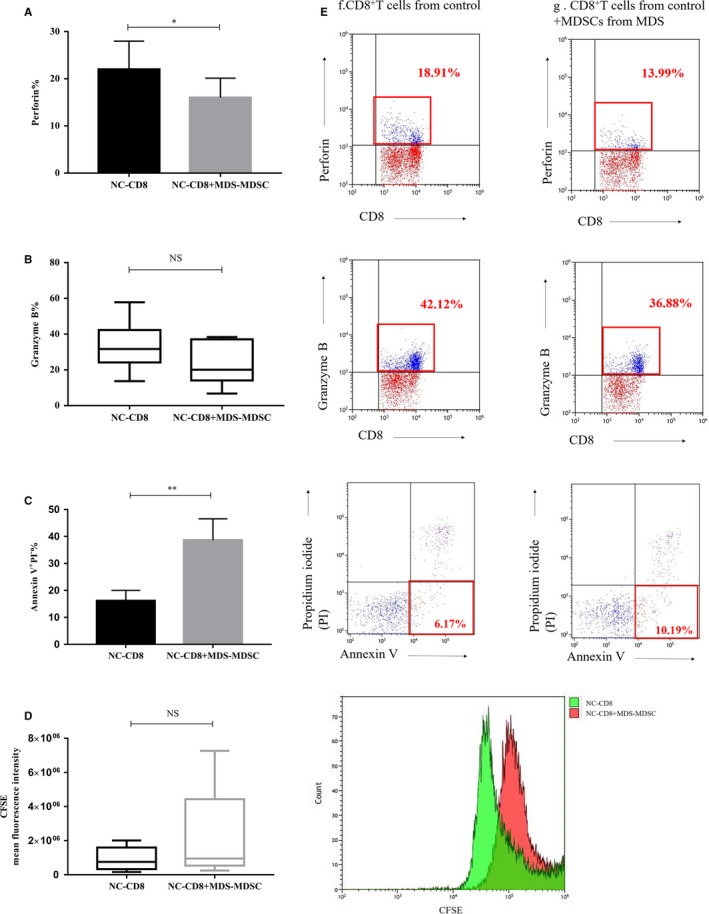
(A‐D) The histogram represents perforin, granzyme B, early apoptosis and the MFI of CFSE in CD8^+^ T cells from normal controls (NC‐CD8) co‐cultured with MDSCs from MDS patients (MDS‐MDSC) or cultured alone. (E) The cells in the red rectangle represent the expression of perforin, granzyme B and early apoptosis of CD8^+^ T cells from NC that were cultures alone or co‐cultures with MDSCs from MDS patients. The red flow histogram represents proliferation of CFSE‐labelled NC‐CD8 co‐cultured with MDS‐MDSC, and the green histogram represents NC‐CD8 cultured alone. *P < .05, **P < .01, NS = not significant

To demonstrate the inhibitory effects of MDSCs on immune cells, we co‐cultured CD8^+^ T cells from NC with MDSCs from MDS patients and analysed perforin and granzyme B (killer cytokine of CD8^+^ T cells) by flow cytometry. The expression of perforin and granzyme B in CD8^+^ T cells from NC co‐cultured with MDSCs from MDS patients (n = 9, samples: 038‐046) was lower than that of CD8^+^ T cells from NC that were cultured alone (perforin: 16.02 ± 5.86% vs 22.02 ± 5.96%, *P* = .0271*; granzyme B: 20.1% (14.08%‐37.12%) vs 31.68% (24.17%‐42.32%), *P* = .0605) (Figure 2A,B). The early apoptosis of CD8^+^ T cells from NC co‐cultured with MDSCs from MDS patients (n = 8, samples: 039‐046) was significantly higher than that of CD8^+^ T cells from NC that were cultured alone (38.66 ± 7.91% vs 16.18 ± 3.84%, *P* = .0075**) (Figure 2C). In order to examine whether the MDSCs in MDS could inhibit the proliferation of CD8^+^ T cells in NC, CD8^+^ T cells from NC were labelled with CFSE and subsequently cultured in the presence or absence of MDSCs from MDS patients (n = 5, samples: 045‐049). The mean fluorescence intensity (MFI) of CFSE in the co‐cultured group showed a slight increase compared with that of the CD8^+^ T cells cultured alone (953075 (533581‐4433061) vs 757364 (330311‐1596830), *P* > .05) (Figure 2D); there was an upward trend in co‐cultured group as seen in the flow cytogram, although there was no significant difference(Figure 2E).

### 3.3 Perforin, granzyme B and early apoptosis of CD8^+^ T cells from MDS after co‐cultivation with MDSCs

#### 3.3.1 Perforin and granzyme B decreased in CD8^+^ T cells from MDS patients after co‐cultivation with MDSCs, which was partially restored by TIM3/Gal‐9 pathway inhibitors

**Figure 3:**
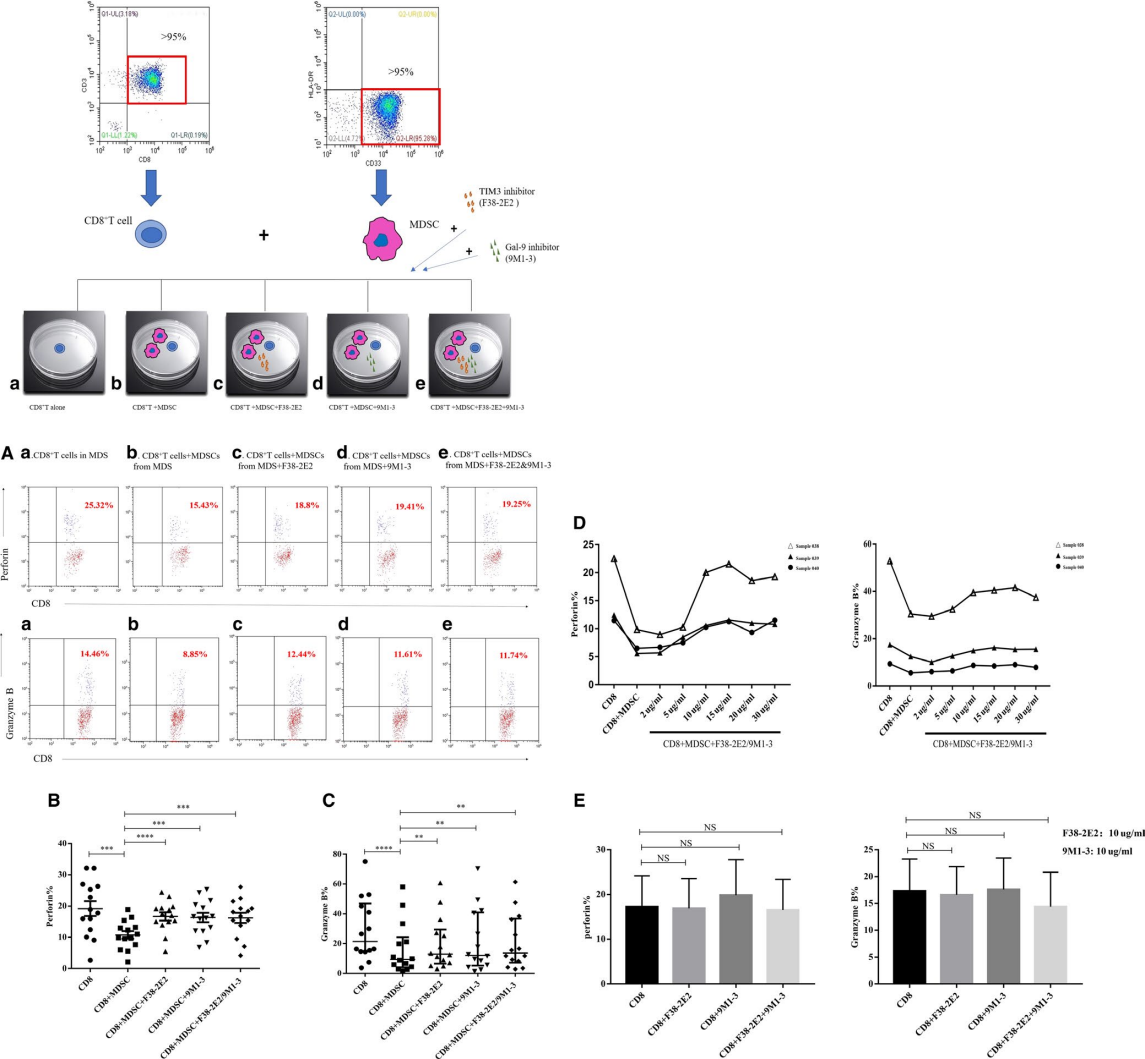
Perforin and granzyme B in CD8^+^ T cells from MDS patients after co‐culture with MDSCs or TIM3/Gal‐9 inhibitors. a: CD8^+^ T cells alone; b: CD8^+^ T cells co‐cultured with MDSCs; c: CD8^+^ T cells+MDSCs+TIM3 inhibitor (F38‐2E2); d: CD8^+^ T cells+MDSCs+Gal‐9 inhibitor (9M1‐3); e: CD8^+^ T cells+MDSCs+F38‐2E2+9M1‐3. (A) The upper right quadrant represents the expression of perforin and granzyme B in CD8^+^ T cells from MDS patients in the five groups. (B) The expression of perforin in the five groups. (C) The expression of granzyme B in the five groups. (D) Effects of different concentrations (2 µg/mL, 5 µg/mL, 10 µg/mL, 15 µg/mL, 20 µg/mL and 30 µg/mL) of inhibitors on perforin and granzyme B expression in CD8^+^ T cells in three patients (sample 038, 039, and 040). (E) Perforin and granzyme B expression in CD8^+^ T cells that were cultured without MDSCs in the presence of inhibitors. *P < .05, **P < .01, ***P < .001, ****P < .0001, NS = not significant

We hypothesized that MDSCs might suppress CD8^+^ T cells through TIM3/Gal‐9 signalling pathway. Therefore, we purified CD8^+^ T cells from MDS patients and co‐cultured them with MDSCs, F38‐2E2 (TIM3 inhibitor) and 9M1‐3 (Gal‐9 inhibitor) respectively. When the concentration of inhibitor was 10 µg/mL, an obvious effect was observed (Figure 3D**)**. The median perforin and granzyme B in group b were significantly lower compared to that of groups a, c, d and e in 14 samples (numbers: 003‐005, 024‐028 and 035‐040) (perforin%: b: 10.76 ± 1.22% vs a: 19.18 ± 2.37% vs c: 16.68 ± 1.33% vs d: 16.35 ± 1.49% vs e: 16.23 ± 1.61%, *P* < .0001****; granzyme B%: b: 9.35% (4.02%‐24.21%) vs a: 21.43% (14.59%‐46.90%) vs c: 12.94% (6.52%‐29.45%) vs d: 12.11% (5.20%‐40.99%) vs e: 13.66% (7.18%‐36.79%), *P* < .0001****) (Figure 3A‐C). The inhibitors had no effect on CD8^+^ T cells cultured alone (Figure 3E).

#### 3.3.2 The early apoptosis of CD8^+^ T cells increased after co‐cultivation with MDSCs and was partially restored by blocking the TIM3/Gal‐9 pathway

**Figure 4:**
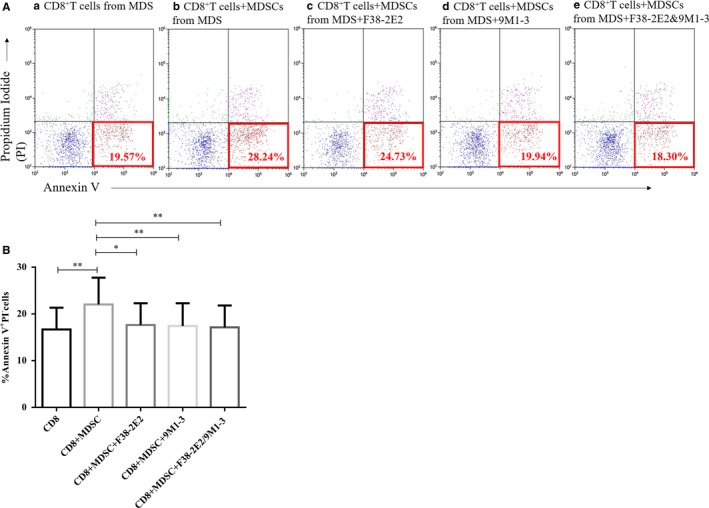
Early apoptosis of CD8^+^ T cells from MDS patients after co‐culture with MDSCs or TIM3/Gal‐9 inhibitors. (A) Scattered dots in the red rectangle represent early apoptosis of CD8^+^ T cells from MDS patients in the five co‐culture systems. The a. b. c. d. and e. experimental groups represent CD8^+^ T cells from MDS, CD8^+^ T cells+MDSCs from MDS, CD8^+^ T cells+MDSCs+F38‐2E2, CD8^+^ T cells+MDSCs+9M1‐3 and CD8^+^ T cells+MDSCs+F38‐2E2+9M1‐3, respectively. (B) The histogram represents early apoptotic cells. *P < .05, **P < .01, NS = not significant

We detected early apoptosis of CD8^+^ T cells from 11 MDS patients (numbers: 003‐005, 027‐031 and 038‐040) after co‐culture with MDSCs and found that early (Annexin V^+^ PI^−^) apoptosis was higher than that of CD8^+^ T cells alone. Furthermore, early apoptosis was reduced after co‐culture with the TIM3 inhibitor (F38‐2E2) and Gal‐9 inhibitor (9M1‐3) (early apoptotic cells%: b: 22.04 ± 5.72% vs a: 16.7 ± 4.64% vs c: 17.63 ± 4.67% vs d: 17.43 ± 4.85% vs e: 17.15 ± 4.66%, *P* = .0002***) (Figure 4A,B).

### 3.4 Decreased perforin and granzyme B in CD8^+^ T cells after co‐cultivation with excess exogenous Gal‐9

We speculated that MDSCs might be a source of Gal‐9 due to its overexpression; therefore, we took excess exogenous Gal‐9 in the place of MDSCs and co‐cultured CD8^+^ T cells with excess exogenous Gal‐9 (recombinant human Gal‐9 [R&D Systems]) and TIM3/Gal‐9 pathway inhibitors.

**Figure 5:**
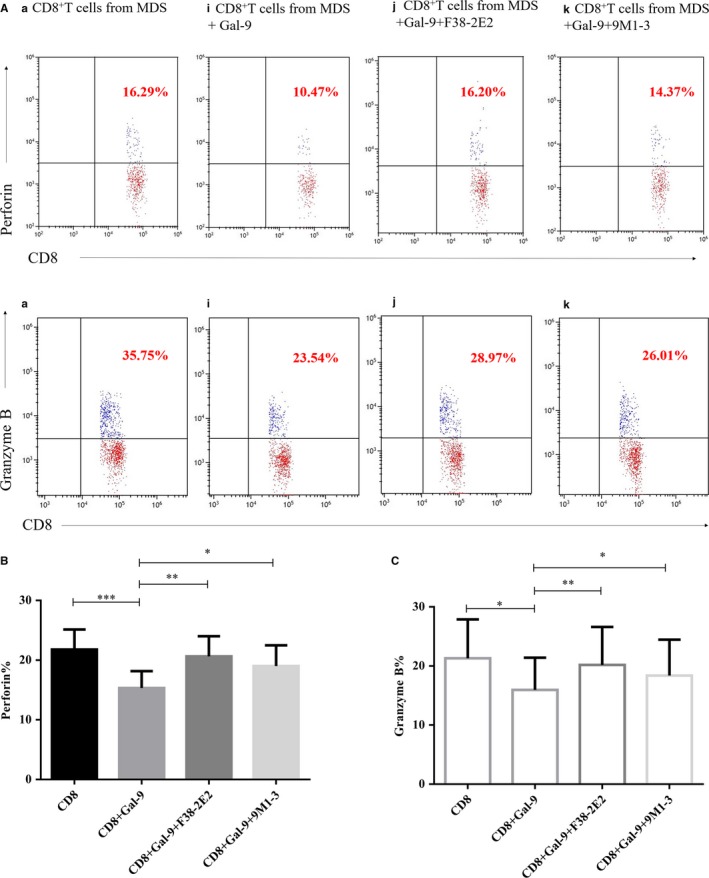
Perforin and granzyme B in CD8^+^ T cells from MDS patients after co‐culture with excess exogenous Gal‐9 or TIM3/Gal‐9 inhibitors. (A) Flow cytometry scatter diagrams of perforin and granzyme B expression in CD8^+^ T cells from MDS. a: CD8^+^ T cells alone; i: CD8^+^ T cells with excess exogenous Gal‐9; j: CD8^+^ T cells from MDS with excess exogenous Gal‐9 and F38‐2E2; and k: CD8^+^ T cells from MDS with excess exogenous Gal‐9 and 9M1‐3. (B and C) The histogram represents the expression of perforin and granzyme B in the four groups. *P < .05, **P < .01, ***P < .001, NS = not significant

The perforin and granzyme B in CD8^+^ T cells purified from MDS patients (n = 9, samples: 026‐028, 034, 036, 037 and 045‐047) were significantly lower after co‐cultivation with excess exogenous Gal‐9 than those in CD8^+^ T cells alone and were partially restored by TIM3 inhibitor or Gal‐9 inhibitor (perforin%: i: 15.33 ± 2.83% vs a: 21.79 ± 3.35% vs j: 20.63 ± 3.37% vs k: 19.01 ± 3.49%, *P* < .0001****; granzyme B%: i: 15.95 ± 5.47% vs a: 21.32 ± 6.56% vs j: 20.18 ± 6.44% vs k: 18.38 ± 6.07%, *P* = .0019**) (Figure 5A‐C).

### 3.5 Notch1, EOMES, p‐mTOR and p‐AKT decreased in CD8^+^ T cells after co‐cultivation with excess exogenous Gal‐9

**Figure 6:**
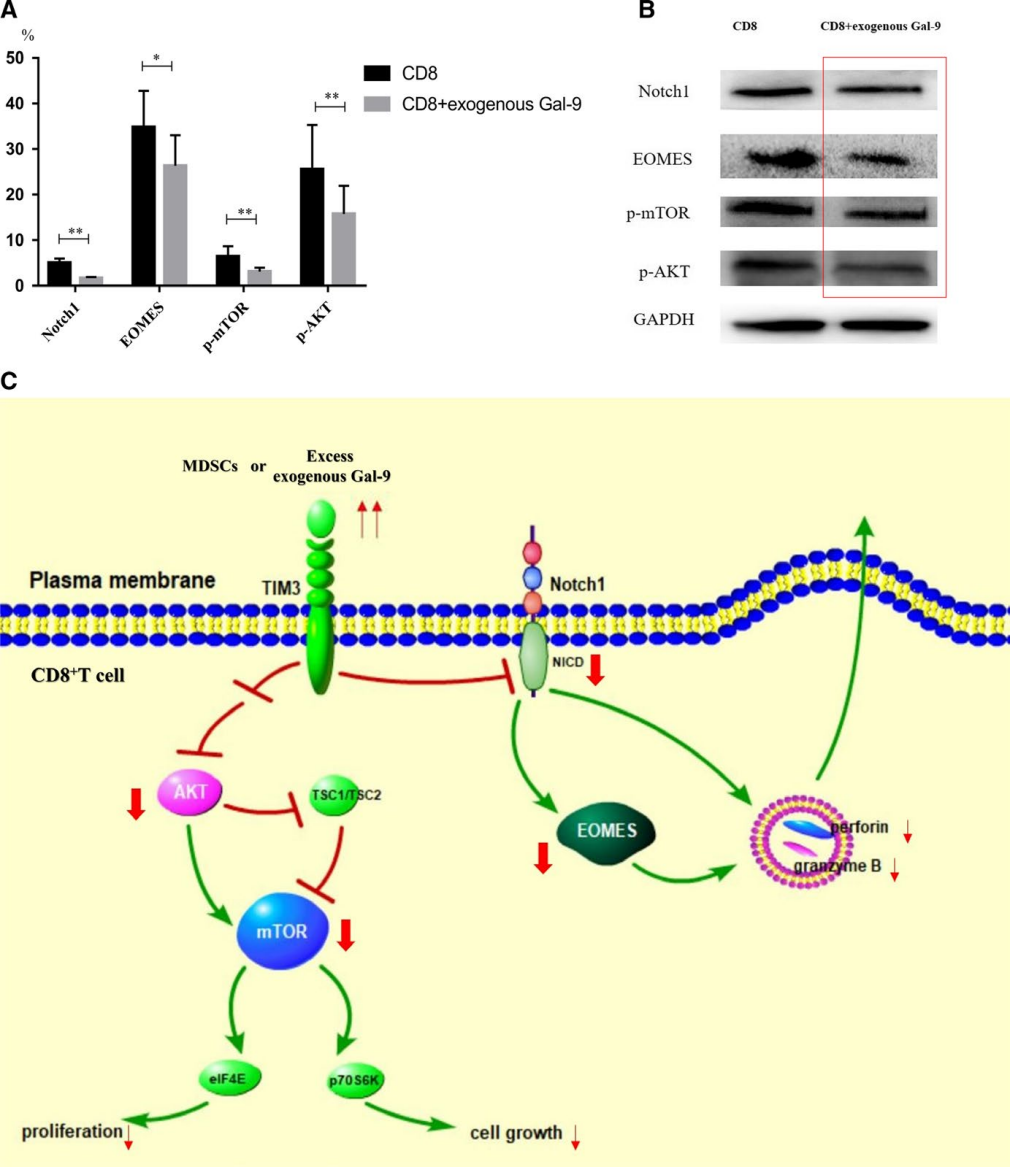
The expression levels of Notch1, EOMES, phospho‐mTOR and phospho‐AKT proteins were reduced in CD8^+^ T cells after co‐culture with exogenous Gal‐9. (A) The histogram representing the expression of Notch1, EOMES, p‐mTOR and p‐AKT by flow cytometry. (B) The expression of Notch1, EOMES, p‐mTOR and p‐AKT by Western blotting. (C) The possible pathogenesis of CD8^+^ T cells from MDS patients' suppression by MDSCs or excess exogenous Gal‐9. *P < .05; **P < .01

We further explored the mechanism of CD8^+^T cell suppression by excess exogenous Gal‐9. The transcriptional regulator EOMES plays a critical role in the development and maturation of CTL. Notch1 regulates EOMES by directly binding to the promoter of key effector molecules and then regulates perforin and granzyme B via EOMES. The expression of Notch1 and EOMES in CD8^+^ T cells (n = 9, samples: 027‐037, except 035,036) treated with excess exogenous Gal‐9 was significantly reduced compared to those of CD8^+^ T cells alone (Notch1: 5.01 ± 0.98% vs 1.7 ± 0.24%, *P* < .01**; EOMES: 34.81 ± 7.96% vs 26.37 ± 6.65%, *P* < .05*) (Figure 6A).

The mammalian target of rapamycin (mTOR) is a serine/threonine (Thr)‐protein kinase that plays a key role in regulating cell growth and proliferation. The expression of p‐mTOR and p‐AKT in CD8^+^ T cells (n = 9, samples: 027‐037, except 035,036) treated with excess exogenous Gal‐9 was lower compared to those of CD8^+^ T cells alone (p‐mTOR: 6.44 ± 2.30% vs 3.15 ± 0.88%, *P* < .01**; p‐AKT: 25.57 ± 9.73% vs 15.78 ± 6.21%, *P* < .01**, Figure 6A). A downward trend in Notch1, EOMES, p‐mTOR and p‐AKT was observed in CD8^+^ T cells after co‐cultivation with excess exogenous Gal‐9 by Western blotting (Figure 6B).

## 4 DISCUSSION

T cell exhaustion, a state of T cell dysfunction characterized by diminished cytokine production and elevated apoptosis, was first characterized in the settings of chronic lymphocytic choriomeningitis virus (LCMV) infection.^18^ T cell exhaustion could also occur in viral infections such as hepatitis C virus and HIV^19^, ^20^ as well as in tumour models^11^, ^21^ and haematological malignant diseases.^22^ The exhausted T cells highly express immune checkpoints, such as TIM3, PD‐1 and CTLA‐4.^6^, ^23^ Blocking TIM3 and PD‐1 can reverse T cell exhaustion and dysfunction in malignant tumour patients^21^, ^24^, ^25^ or neoplastic mice.^26^ These data suggested that CD8^+^ T cells exhaustion in human or mice could be induced by immune checkpoints in the setting of chronic viral infection or malignant diseases.

Increasing malignant clones and cellular immunity defects are involved in the pathogenesis of MDS,^1^, ^2^, ^3^, ^4^, ^5^ which is a malignant haematological disease. Daver et al^27^ found that the efficacy of hypomethylating agent combination with immune checkpoint inhibitors (PD‐1 inhibitor) in MDS was better than hypomethylating agents alone. TIM3, as one of the important immunity checkpoints, interacts with its ligand Gal‐9^24^ could promote Tc1 cell^9^ apoptosis, CD8^+^T cell exhaustion,^10^, ^11^ malignant cell proliferation^12^ and MDSC proliferation.^13^ MDSCs, which are powerful cellular immunity suppressor, could badly inhibit the antitumour immune response mediated by the effector T cells and lead to tumour cells immunological surveillance escape.^28^, ^29^

Here, we found that Gal‐9 was also highly expressed in MDSCs from MDS patients and was increasing in the bone marrow supernatant, serum and MDSC culture supernatants. These data suggested that MDSCs could secrete Gal‐9 into the microenvironment and exert an immunosuppressive effect. Hayashi et al^30^ found that inflammatory pathway proteins that regulate immunity were moderately elevated in both low‐risk and high‐risk MDS patients but were much lower than that in real infectious diseases. These suggested that cellular immunity was insufficient for malignant clonal cells due to cellular immunity tolerance, both in low‐risk and high‐risk MDS groups. We found that there was no difference of Gal‐9 in MDSCs of high‐risk and low‐risk MDS patients, which was consistent with our hypothesis. Therefore, further exploration of the molecular mechanism of Gal‐9 up‐regulation is needed.

Perforin and granzyme B are the most important cytokines in CD8^+^ T cells against infectious and malignant cells. MDSCs can inhibit CD8^+^ T cells in esophageal squamous cell carcinoma.^8^ Here, perforin, granzyme B and proliferation decreased and the early apoptosis of CD8^+^ T cells increased after CD8^+^ T cells from controls were co‐cultured with MDSCs. These data implied that MDSCs with excessive Gal‐9 might induce TIM3 overexpression in CD8^+ ^T cells, leading to a downward trend of perforin and granzyme B after CD8^+^ T cells from MDS were co‐cultured with MDSCs. We hypothesized that MDSCs might be a source of Gal‐9. Therefore, we replaced MDSCs with excessive exogenous Gal‐9 to repeat the above procedure and found that CD8^+^ T cells indeed produced less perforin and granzyme B after co‐cultivation with excessive exogenous Gal‐9, similar to that after co‐cultivation with MDSCs from MDS patients. The secretion of perforin and granzyme B by CD8^+^ T cells could be partially restored by TIM3/Gal‐9 pathway inhibitors.

Meanwhile, the early apoptosis of CD8^+^ T cells after co‐culture with MDSCs was higher than that of CD8^+^ T cells alone and could be partially restored by TIM3/Gal‐9 pathway inhibitors. It suggested that CD8^+^ T cells exhaustion might be induced by MDSC through TIM3/Gal‐9 pathway.

The T‐box transcription factor eomesodermin (EOMES) plays an important role in the development and maturation of CTL.^31^ Notch1 could regulate perforin and granzyme B by affecting EOMES.^15^, ^32^ Here, we found that Notch1 and EOMES decreased after co‐cultivation with excessive exogenous Gal‐9. It indicated that the TIM3/Gal‐9 pathway might induce CD8^+^T cells ‘exhaustion’ by down‐regulating Notch1 and EOMES.

Mammalian target of rapamycin (mTOR) is a serine/threonine (Thr)‐protein kinase that plays a central role in regulating cell growth and proliferation.^33^ Overactivation of AKT/mTOR after phosphorylation is associated with over‐proliferation of leukaemia cells,^16^, ^34^ as well as tumour cells such as liver cancers and pancreatic neuroendocrine tumours.^35^, ^36^ Our study found that p‐mTOR and p‐AKT decreased in CD8^+^ T cells after co‐culture with exogenous Gal‐9, indicating that TIM3/Gal‐9 pathway might suppress CD8^+^ T cells through down‐regulating AKT/mTOR (Figure 6C).

In conclusion, CD8^+^ T cells ‘exhaustion’ could be induced by up‐regulating Gal‐9 in MDSCs and TIM3 in CD8^+^ T cells of MDS patients, which was partially restored by TIM3/Gal‐9 pathway inhibitors. TIM3/Gal‐9 pathway may be involved in CD8^+^ T cells exhaustion induced by MDSCs in MDS, leading to malignant MDS clone immunological surveillance escape and over‐proliferation. Therefore, TIM3/Gal‐9 pathway inhibitors might be the promising candidate for target therapy of MDS in the future.

## CONFLICT OF INTEREST

The authors confirm that there are no conflicts of interest.

## **AUTHORS**'** CONTRIBUTIONS**

Tao Jinglian and Han Dong contributed equally to performing research, analysing the data and writing the manuscript; Shao Zonghong, Li Lijuan and Fu Rong designed research, and ensured correct analysis of the data, and contributed to the writing of the manuscript; and Gao Shan, Zhang Wei, Yu Hong and Liu Pei assisted in the designing of research, overseeing the collection of the data and contributed to the writing of the manuscript.

## Data Availability

More detailed data of this study are available from the corresponding author upon request.
